# Development of a versatile synthetic microbe for the concurrent degradation of organic pollutants

**DOI:** 10.1007/s44307-025-00072-w

**Published:** 2025-05-23

**Authors:** Cong Su, Shuangying Jiang, Junbiao Dai

**Affiliations:** 1 Shenzhen Key Laboratory of Synthetic Genomics, Guangdong Provincial Key Laboratory of Synthetic Genomics, Key Laboratory of Quantitative Synthetic Biology, Shenzhen Institute of Synthetic Biology, Shenzhen Institute of Advanced Technology, Chinese Academy of Sciences, Shenzhen, 518055 China; 2 Shenzhen Branch, Guangdong Laboratory for Lingnan Modern Agriculture, Key Laboratory of Synthetic Biology, Ministry of Agriculture and Rural Affairs, Agricultural Genomics Institute at Shenzhen, Chinese Academy of Agricultural Sciences, Shenzhen, China

## Introduction

One of the most formidable challenges in environmental protection is the remediation of complex pollutants containing multiple aromatic compounds. Globally, aromatic compounds, including benzene, toluene, ethylbenzene, and xylene (BTEX), along with their derivatives and polycyclic aromatic hydrocarbons (PAHs), are major contributors to soil and groundwater contamination (Haritashi and Kaushik 2009). Once released into the environment, these compounds exhibit significant resistance to degradation through natural biological metabolism, photocatalysis, and other processes (Davidson et al. 2021). Consequently, they can persist in environments such as soil, water, and sediment for decades to centuries, or potentially even longer. Currently, Su et al. tackle the challenge of persistent organic pollutants by engineering a fast-growing strain of Vmax bacteria. A large synthetic DNA fragment (~ 43 kbp) was stably integrated into the bacterial genome, enabling the degradation of five major classes of organic pollutants (Su et al. 2025).

## Microbial degradation of pollutants

The question of how to effectively degrade these complex pollutants has garnered significant attention from researchers in environmental pollution control. These compounds can be broken down or transformed by bacteria with specific metabolic capabilities (Antizar-Ladislao et al. 2006). However, microbial catabolic enzymes are typically specialized for a single molecule or a limited range of molecules with similar chemical structures (Wang et al. 2013). Historically, native microbiota or artificially constructed consortia, comprising strains capable of degrading diverse compounds, have been employed to remediate environments contaminated with complex organic pollutants, such as PAH-polluted soil, petroleum-contaminated land, or crude oil-polluted seawater (Aiyar et al. 2002).

## When bioremediation meets synthetic biology

This study was driven by a critical question: Can a single strain be engineered to harbor multiple degradation gene clusters and simultaneously degrade various pollutants? In recent years, microbial remediation has garnered increasing attention, especially within the emerging field of synthetic biology. Synthetic biology offers powerful tools to engineer microbes capable of sensing, aggregating, and degrading environmental toxins, thereby contributing to the remediation of polluted water, soil, and air (Huang et al. 2022). Based on this rationale, we proposed the development of a synthetic strain, through synthetic biology approaches, capable of degrading multiple pollutants in saline environments.

## Advantages of Vmax as a chassis

*Vibrio natriegens* Vmax is the fastest-growing non-pathogenic organism known to date, with a generation time of less than 10 min. This bacterium exhibits broad metabolic capabilities, high substrate uptake rates, exceptional tolerance to high salt concentrations, and an absence of pathogenicity. Furthermore, it is genetically amenable and capable of efficiently expressing foreign proteins, making it an attractive and robust chassis for applications in biotechnology and synthetic biology. *V. natriegens* possesses a rich array of genome editing tools, including double-crossover homologous recombination, the Cre-loxP system, and others, further enhancing its utility as a versatile platform organism (Weinstock, et al. 2016).

## Development of an optimal transformation method

*V. natriegens* can undergo natural transformation(NT), enabling it to become competent under relatively mild conditions without the need for electroporation or chemical treatments. It can absorb external DNA and integrates it into its chromosome. Multiplex Genome Editing by Natural Transformation (MuGENT), a multiplex genome editing technique customized for *V. natriegens*, enables efficient multi-gene integration (Dalia et al. 2017). These tools and features make *V. natriegens* a promising new chassis for molecular cloning and biotechnology applications. Based on the previous research, the *tfoX* gene from *V*. *cholerae* was inserted into the chromosome of *V*. *natriegen* to enhance its natural transformation efficiency, which was ultimately improved by 5- to 10- fold.

## Application of the engineered strain

To address the challenges of industrial wastewater bioremediation, VCOD-15, an artificial microorganism containing five synthetic gene clusters (43 kbp) derived from different species, was constructed. It demonstrates robust enzymatic activity in chlor-alkali wastewater with salinity as high as 102.5 g per liter, effectively overcoming the limitation of traditional strains that typically"lose activity upon exposure to salt."Within an activated sludge reactor, high-concentration pollutants were completely degraded within 12 h. Testing across multiple parallel bioreactors revealed that, within 48 h, the residual pollutant levels in the industrial wastewater were consistently below 2% of the detection limit, while the proportion of VCOD-15 in the complex microbial community remained stable at over 40%. These results highlight its exceptional environmental adaptability and competitive dominance.

## Artificial bacterial community vs engineered strains

**Fig. 1:**
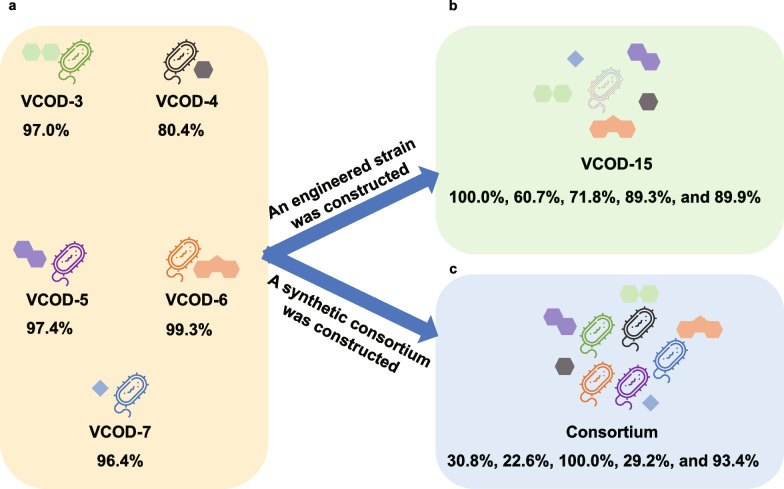
The degradation efficiencies of different degradation strains of biphenyl, phenol, naphthalene, dibenzofuran and toluene. **a** The degradation efficiency of the engineered strains VCOD-3, VCOD-4, VCOD-5, VCOD-6, and VCOD-7. **b** The degradation efficiency of the engineered strain VCOD-15. **c** The degradation efficiency of the artificial community constructed by 5 engineered strains (VCOD-3, VCOD-4, VCOD-5, VCOD-6, and VCOD-7)

In this study, five additional engineered strains (VCOD-3, VCOD-4, VCOD-5, VCOD-6, and VCOD-7) were successfully constructed, each capable of degrading a specific aromatic compound. This finding indicates the potential for constructing an artificial microbial consortium to degrade complex pollutants. The synthetic community exhibited degradation efficiencies of 30.8% for biphenyl, 22.6% for phenol, 100% for naphthalene, 29.2% for dibenzofuran, and 93.4% for toluene. However, the degradation rates of several pollutants, particularly those of biphenyl, phenol, and toluene, were significantly lower than those achieved by individual strains and also lower than the performance of the engineered strain VCOD-15 (Fig. 1). These results underscore the need for further optimization of artificial microbial consortia to improve cooperative degradation efficiency.

## Conclusion

Compared to conventional strains engineered for single-pollutant degradation, Su et al. utilized synthetic biology approaches to integrate multiple rationally designed gene clusters, targeting naphthalene, phenanthrene, phenol, and halogenated hydrocarbons, into a halotolerant chassis strain. This engineered strain enhances metabolic compatibility through optimization, thereby overcoming substrate specificity limitations and enabling efficient simultaneous degradation of aromatic and halogenated hydrocarbons. Notably, this strain demonstrates exceptional performance in hypersaline environment contaminated with complex pollutants.
